# Rare and common epilepsies converge on a shared gene regulatory network providing opportunities for novel antiepileptic drug discovery

**DOI:** 10.1186/s13059-016-1097-7

**Published:** 2016-12-13

**Authors:** Andree Delahaye-Duriez, Prashant Srivastava, Kirill Shkura, Sarah R. Langley, Liisi Laaniste, Aida Moreno-Moral, Bénédicte Danis, Manuela Mazzuferi, Patrik Foerch, Elena V. Gazina, Kay Richards, Steven Petrou, Rafal M. Kaminski, Enrico Petretto, Michael R. Johnson

**Affiliations:** 1 Division of Brain Sciences, Imperial College Faculty of Medicine, London, UK; 2 MRC Clinical Sciences Centre, Imperial College London, London, UK; 3 Université Paris 13, Sorbonne Paris Cité, UFR de Santé, Médecine et Biologie Humaine, Paris, France; 4 PROTECT, INSERM, Université Paris Diderot, Sorbonne Paris Cité, Paris, France; 5 Duke-NUS Medical School, 8 College Road, 169857 Singapore, Republic of Singapore; 6 Neuroscience TA, UCB Pharma, S.A, Allée de la Recherche, 60, 1070 Brussels, Belgium; 7 The Florey Institute of Neuroscience and Mental Health, The University of Melbourne, Parkville, Victoria 3052 Australia; 8 The Centre for Neural Engineering, The Department of Electrical Engineering, The University of Melbourne, Parkville, Victoria 3052 Australia; 9 The Australian Research Council Centre of Excellence for Integrative Brain Function, Parkville, Victoria 3052 Australia

**Keywords:** Epilepsy, Systems genetics, Co-expression, Regulatory network, Protein-protein interactions, Epileptic encephalopathy, SCN1A, Valproic acid

## Abstract

**Background:**

The relationship between monogenic and polygenic forms of epilepsy is poorly understood and the extent to which the genetic and acquired epilepsies share common pathways is unclear. Here, we use an integrated systems-level analysis of brain gene expression data to identify molecular networks disrupted in epilepsy.

**Results:**

We identified a co-expression network of 320 genes (M30), which is significantly enriched for non-synonymous de novo mutations ascertained from patients with monogenic epilepsy and for common variants associated with polygenic epilepsy. The genes in the M30 network are expressed widely in the human brain under tight developmental control and encode physically interacting proteins involved in synaptic processes. The most highly connected proteins within the M30 network were preferentially disrupted by deleterious de novo mutations for monogenic epilepsy, in line with the centrality-lethality hypothesis. Analysis of M30 expression revealed consistent downregulation in the epileptic brain in heterogeneous forms of epilepsy including human temporal lobe epilepsy, a mouse model of acquired temporal lobe epilepsy, and a mouse model of monogenic Dravet (*SCN1A*) disease. These results suggest functional disruption of M30 via gene mutation or altered expression as a convergent mechanism regulating susceptibility to epilepsy broadly. Using the large collection of drug-induced gene expression data from Connectivity Map, several drugs were predicted to preferentially restore the downregulation of M30 in epilepsy toward health, most notably valproic acid, whose effect on M30 expression was replicated in neurons.

**Conclusions:**

Taken together, our results suggest targeting the expression of M30 as a potential new therapeutic strategy in epilepsy.

**Electronic supplementary material:**

The online version of this article (doi:10.1186/s13059-016-1097-7) contains supplementary material, which is available to authorized users.

## Background

Epilepsy is a common, serious neurological disease principally characterised by a tendency to recurrent unprovoked epileptic seizures [1–4]. Despite a large number of antiepileptic drugs (AEDs) currently available to treat epilepsy, approximately one-third of people with epilepsy have continuing uncontrolled seizures and there remains a global imperative to develop new therapeutic approaches [5].

Traditional twin-based estimates of heritability [6–8] and more recent genomic heritability analyses [9] have established that epilepsy has a substantial genetic component. Idiopathic epilepsy arising from an identified or presumed genetic etiology is termed ‘genetic epilepsy’ [10]. Analysis of genetic epilepsy segregating in a Mendelian fashion using traditional linkage analysis led to the identification of several genes for epilepsy (reviewed in [11, 12]). The majority of these Mendelian idiopathic epilepsy genes encode ion channel subunits leading to the concept of the genetic epilepsies as ‘ion channelopathies’. More recently, the application of next-generation sequencing to the epileptic encephalopathies (EE), a group of severe childhood-onset epilepsies associated with refractory seizures and intellectual disability (ID), have underscored the importance of synaptic dysfunction in epilepsy and established de novo mutagenesis as a major genetic mechanism for EE [13, 14].

Common variant contributions to the more common forms of adult and childhood epilepsy are less well defined, although a recent meta-analysis of genome-wide association studies (GWAS) in genetic generalised and focal epilepsy identified genome-wide significant variants in two genes involving synaptic function, *SCN1A* and *PCDH7* [15]. Analysis of genetic contributions to these presumed polygenic forms of epilepsy using genotypes recorded over single nucleotide polymorphisms (SNPs) revealed that common variants collectively explain substantial phenotypic variation of epilepsy and suggested that at least 400 variants (and potentially many thousands) influence disease susceptibility [9]. For these common epilepsies, there is an unresolved debate about whether genetic susceptibility arises as a result of polygenic contributions from common variants or whether these epilepsies comprise a large number of discrete diseases arising from rare monogenic variation tagged by SNPs (reviewed in [9, 16]).

As well as genetic contributions to epilepsy, in approximately 25% of cases the epilepsy has a clearly defined acquired cause, such as following status epilepticus (SE), head trauma or stroke [17]. While the precise mechanisms underpinning the development of epilepsy following brain injury (a process termed ‘epileptogenesis’) are poorly understood, changes in expression of ion channels genes and synaptic receptors have been reported [18], leading to the proposal that acquired epilepsy (AE) may be a secondary ion channelopathy. These observations suggest a possible convergence of mechanisms for genetic and acquired epilepsy.

Systems biology and network analyses provide powerful approaches to elucidate the molecular processes and pathways underlying disease [19, 20]. Using genome-wide transcriptional profiling in tissues relevant to the disease under investigation, gene co-expression network analysis can identify gene modules (i.e. sets of co-expressed genes) as candidate regulators and drivers of disease [21, 22]. The assumption is that the modular structure of co-expression reflects the underlying activity of shared regulatory mechanisms among sets of genes encoding functionally related proteins [23]. Applications of co-expression network methodology to epilepsy to date have identified a pro-convulsant inflammatory gene network in the human epileptic hippocampus [24] and revealed overlap between genes that cause epileptic encephalopathy when mutated and those that contribute to variation in healthy human intelligence [25]. Beyond a better understanding of molecular drivers of disease, it is increasingly the case that network analysis can also provide new candidate targets for drug discovery or repurposing [26, 27].

Here, for the first time, we used a systems-level approach based on gene co-expression network analysis of the healthy human brain to identify physiological processes and pathways perturbed in epilepsy. We aimed to address a number of currently unanswered questions, including whether there are brain-region specific contributions to epilepsy, and the relationship between gene networks for genetic epilepsy and molecular processes disrupted in acquired epilepsy. The overall goal of our study was to discover and prioritise gene networks that could be targeted for future AED development.

**Fig. 1:**
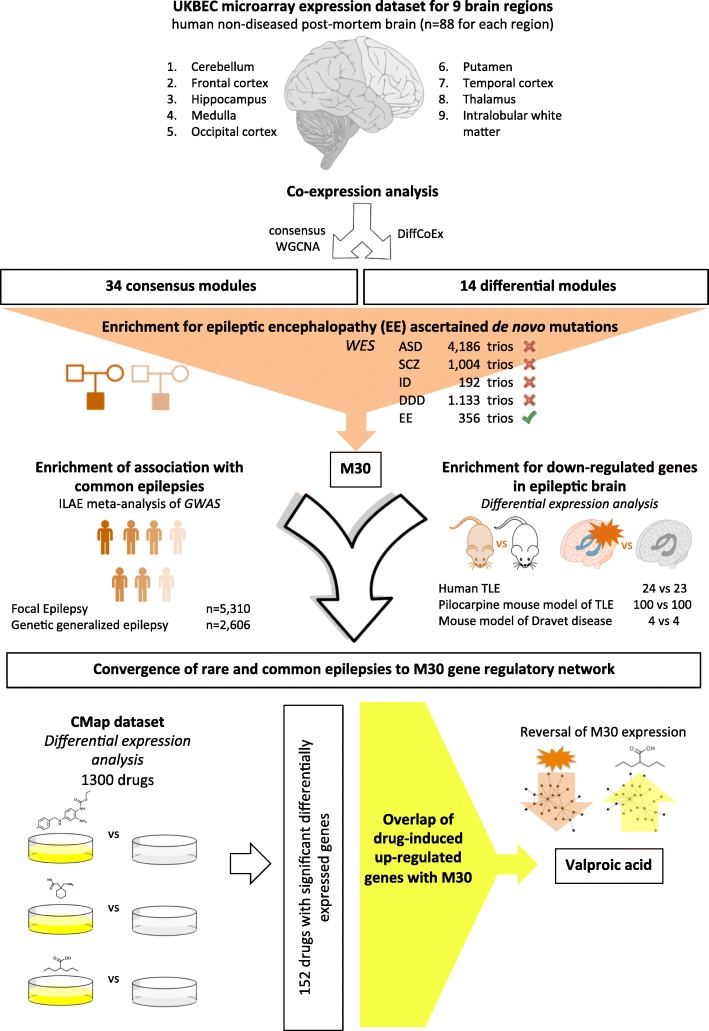
*Schematic overview* of study design. We hypothesised that gene regulatory networks in the human healthy brain disrupted by de novo mutations ascertained from patients with epileptic encephalopathy could be informative for molecular processes involved in different types of epilepsy. We used post-mortem human brain samples ascertained from individuals with no history of psychiatric or neurological illness from the UK Brain Expression Consortium (UKBEC) dataset to build gene co-expression networks (modules) that are expressed across the whole brain (‘consensus’ modules, using the WCGNA consensus method), or differential to one brain region or another (‘differential’ modules, using DiffCoEx method). In order to prioritise modules relevant to epilepsy, we integrated modules with whole-exome-sequencing (WES) studies data of rare de novo mutations ascertained from patients with epileptic encephalopathy (EE) and neurodevelopmental disease more generally (*ASD* autism spectrum disorder, *SCZ* schizophrenia, *ID* intellectual disabilities, *DDD* broad developmental disease from the Deciphering Developmental Disorders study). A single module was selected in this way: M30. Our hypothesis was that disruption of this gene network might lead to different types of epilepsy. M30 was therefore tested for enrichment of association to common forms of epilepsy using GWAS data from the International League against Epilepsy (ILAE) meta-analysis. Analysis of network genes’ expression in disease in three epilepsies suggested functional disruption and/or downregulation of the network as a common mechanism regulating susceptibility to epilepsy broadly and therefore that the network itself might be targeted as a novel therapeutic strategy. As a proof of concept, we show that among the drugs capable of inducing transcriptional changes in cells of the Connectivity map (Cmap) dataset, VPA, a widely used AED with a broad spectrum of clinical efficacy, is the one that is most significantly predicted to restore the expression of M30 in epilepsy toward health. Full details relating to datasets, experimental methods and references are provided in the manuscript

To identify gene-regulatory networks for epilepsy we chose post-mortem brain samples from healthy subjects as our starting point because we wanted to identify normal brain processes perturbed in epilepsy and to avoid the potential confounding effects of secondary or homeostatic changes in gene expression related to the occurrence of seizures themselves. A summary of our experimental design is shown in Fig. 1. Briefly, we used post-mortem human brain samples ascertained from individuals with no history of psychiatric or neurological illness to build gene co-expression networks (modules) that were expressed across the whole brain or differential to one brain region or another. In order to prioritise modules relevant to epilepsy we: (1) integrated modules with a large database of rare de novo mutations ascertained from patients with monogenic EE (and neurodevelopmental disease more generally); and (2) tested modules for enrichment of association to common forms of epilepsy using GWAS data. Utilising this integrative approach, we report a single network of 320 co-expressed genes genetically associated with monogenic and polygenic forms of epilepsy. Further analysis revealed functional disruption and/or downregulation of this network as a common mechanism regulating susceptibility to genetic and acquired epilepsy broadly, suggesting the network itself might be targeted as a novel therapeutic approach. As proof of concept for this, we show that among the many drugs capable of inducing transcriptional changes in cells [28], valproic acid (VPA), a widely used AED with a broad spectrum of clinical efficacy, is the one that is most significantly predicted to restore the expression of the network in epilepsy toward health.

## Results

### Gene co-expression network analysis in the brain

We hypothesised that unsupervised genome-wide co-expression network analysis in the healthy human brain may be informative for functional pathways disrupted in epilepsy. As a first step, we aimed to identify gene co-expression modules that were: (1) specific to an anatomical brain region; or (2) co-expressed widely across the human brain. The relationship of co-expression networks to epilepsy was then investigated using an integrated approach, exploring first the enrichment of co-expression networks for monogenic and polygenic epilepsy genetic risk factors. We then evaluated the relationship of networks genetically associated to epilepsy to acquired forms of epilepsy.

As starting material, we used 88 post-mortem human brains from the UK Brain Expression Consortium (UKBEC) [29], where genome-wide gene expression had been assessed across nine brains regions: cerebellar cortex, temporal cortex, frontal cortex, occipital cortex, hippocampus, thalamus, white matter, medulla, and putamen. Co-expression modules were inferred from these datasets using two approaches: (1) consensus Weighted Gene Co-expression Network Analysis (WGCNA) to identify co-expression modules common to all nine brain regions (‘consensus modules’); and (2) DiffCoEx [30] to construct modules of differentially co-expressed genes in one or several brain regions compared to the other regions (‘differential modules’). This identified 34 consensus modules and 13 differential modules, respectively (see Additional file 1: Table S1).

We then investigated whether these modules were reproducible in separate gene expression datasets. To this end, we used two unrelated publicly available brain expression datasets (see Additional file 2: Table S2) and assessed module conservation using the Z_*summary*_ value [31] (see ‘Methods’). First, we used micro-array gene expression data spanning fetal and post-natal human brain development (‘Brainspan’, GSE25219) [32, 33]; we observed that 32 of 47 (68%) modules were at least moderately preserved (Z_*summary*_ >5) in at least one brain region during fetal development, and that these modules were generally more significantly (Z_*summary*_ >10) preserved in cortical samples from post-natal brain (see Additional file 3: Figure S2).

We then analysed the preservation of co-expression modules using messenger RNA (mRNA)-sequencing (RNA-seq) expression data across different brain regions as well as in non-brain tissues (latter to assess specificity) from the Genotype-Tissue Expression (GTEx) project [34, 35]. We observed that the brain co-expression modules from UKBEC data were generally poorly preserved in non-cortical tissue (e.g. lymphocytes, fibroblasts) (see Additional file 3: Figure S2), but well preserved using GTEx data relating to cortical brain regions; 20 out of 34 (59%) consensus modules and 8 out of the 13 (61%) differential modules were highly significantly preserved (Z_*summary*_ >10) in at least one brain region from both the Brainspan and GTEx datasets. Finally, we assessed module preservation across-species using RNA-seq data from 100 healthy adult mouse hippocampus samples [24]; 20 out of 34 (59%) human consensus modules and 8 out the 14 (57%) differential modules were at least moderately preserved in the healthy mouse hippocampus (Z_*summary*_ >5).

In summary, taking into account the overlap of modules preserved according to these comparative network analyses, we identified 18 consensus and eighy differential modules that are reproducible in unrelated brain expression datasets including between species and across pre- and post-natal human life. We hypothesised that these preserved gene networks inferred from healthy human brain samples represent a transcriptional architecture underpinning critical brain functions and so investigated their relationship to epilepsy.

### Integration with rare de novo epilepsy variants

De novo single nucleotide variants (de novo mutations, DNMs) were collated from published whole-exome sequencing (WES) studies of probands with monogenic EE and their unaffected parents (*n* = 356 trios) [13, 14]. In view of the established genetic overlap between neurodevelopmental disorders [25, 36, 37], we extended the analysis to include WES trio datasets for autism spectrum disorder (ASD, *n* = 4186), schizophrenia (SCZ, *n* = 1004), ID (*n* = 192), and broad developmental disease from the Deciphering Developmental Disorders study (DDD, *n* = 1,133) (see ‘Methods’ for cohort references). For each co-expression module, we tested for the enrichment of DNMs, focusing on non-synonymous DNMs (consisting of all missense, nonsenses, and splice-site mutations) and including tests of enrichment for synonymous DNMs as a negative control. We also calculated enrichment of DNMs among the set of ‘Background’ genes (i.e. all genes significantly expressed in the UKBEC brain regions used to infer the co-expression modules).

**Fig. 2:**
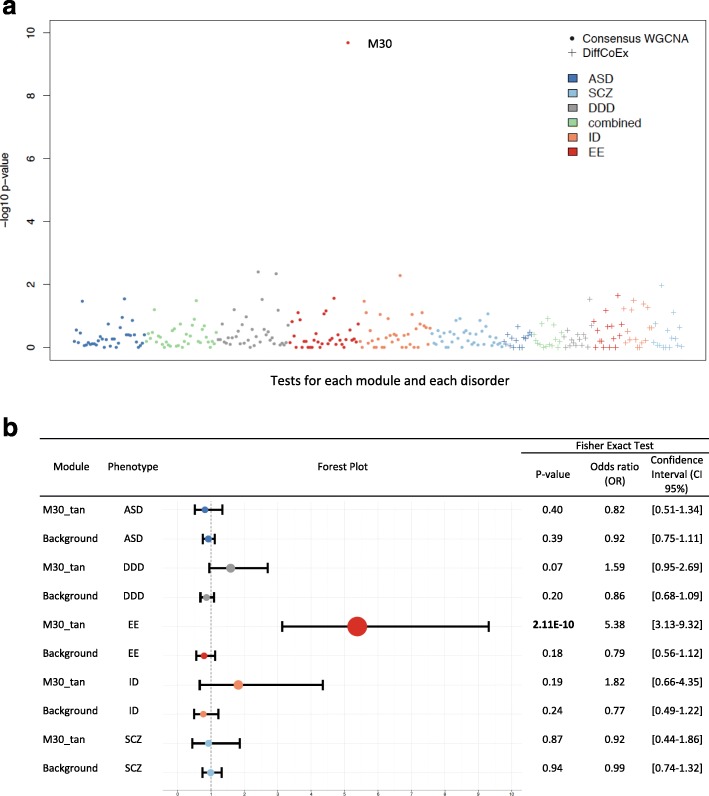
Enrichment of non-synonymous DNM from patients with neurodevelopmental disease. **a** Statistical significance of over-representation of DNM in cases compared to controls for all modules. For each co-expression module obtained from consensus WGCNA (*circle*, *n* = 34) and DiffCoEx (*plus* symbol, *n* = 13), the enrichment was tested adopting FET (see ‘Methods’). Each *dot* represents a module and its significance of enrichment in non-synonymous DNMs (consisting of all missense, nonsenses, and splice-site mutations) is reported on the *y-axis*. The over-representation of DNM in cases compared to controls was calculated for several phenotypes: ASD (4186 trios, *dark blue dots*), SCZ (1004 trios, *light blue dots*), congenital abnormalities of the DDD study (1133 trios, *grey dots*), across four neurodevelopmental disorders consisting of EE, ID, ASD and SCZ (combined, 5738 trios, *green dots*), ID (192 trios, *orange dots*) and EE (356 trios, *red dots*). **b** Enrichment of non-synonymous DNM from patients with neurodevelopmental disease in M30 module. *P* value, OR and 95% CI are reported for M30 and all genes expressed in the UKBEC samples (background). In the *forest plot*, the magnitude of the ORs are represented by the area of the *circles* and the 95% CI by *horizontal lines*

Integrating co-expression modules with DNM data across these five phenotypic categories (EE, ASD, SCZ, ID, DDD), we identified only a single module (consensus module M30), which was highly and specifically enriched for non-synonymous DNMs for EE (Fisher’s exact test (FET) *P* = 2.11 × 10^–10^, odds ratio (OR) = 5.38, 95% confidence interval (CI) = 3.13–9.32). This enrichment remained significant after adjustment for the number of modules and phenotypes tested (Bonferroni corrected *P* value threshold = 6.44 × 10^–8^). No other module was significantly enriched for non-synonymous DNM for any neurodevelopmental phenotype above the Background (see Fig. 2, Additional file 4: Table S3). There was no enrichment for any module in disease-ascertained synonymous DNMs.

Functionally, module M30 is highly enriched for genes relevant to various neural processes, including ‘transmission of nerve impulse’ (Benjamini–Hochberg (BH) corrected *P* = 3.25 × 10^–17^, ratio of enrichment (*r*) = 4.0), ‘synaptic transmission’ (BH *P* = 7.75 × 10^–15^, *r* = 4.0), ‘gamma-aminobutyric acid (GABA) signalling pathway’ (BH *P* = 6.64 × 10^–6^, *r* = 7.56) and ‘synaptic vesicle transport’ (BH *P* = 6.46 × 10^–7^, *r* = 18.37) (see Additional file 1: Table S1).

**Fig. 3:**
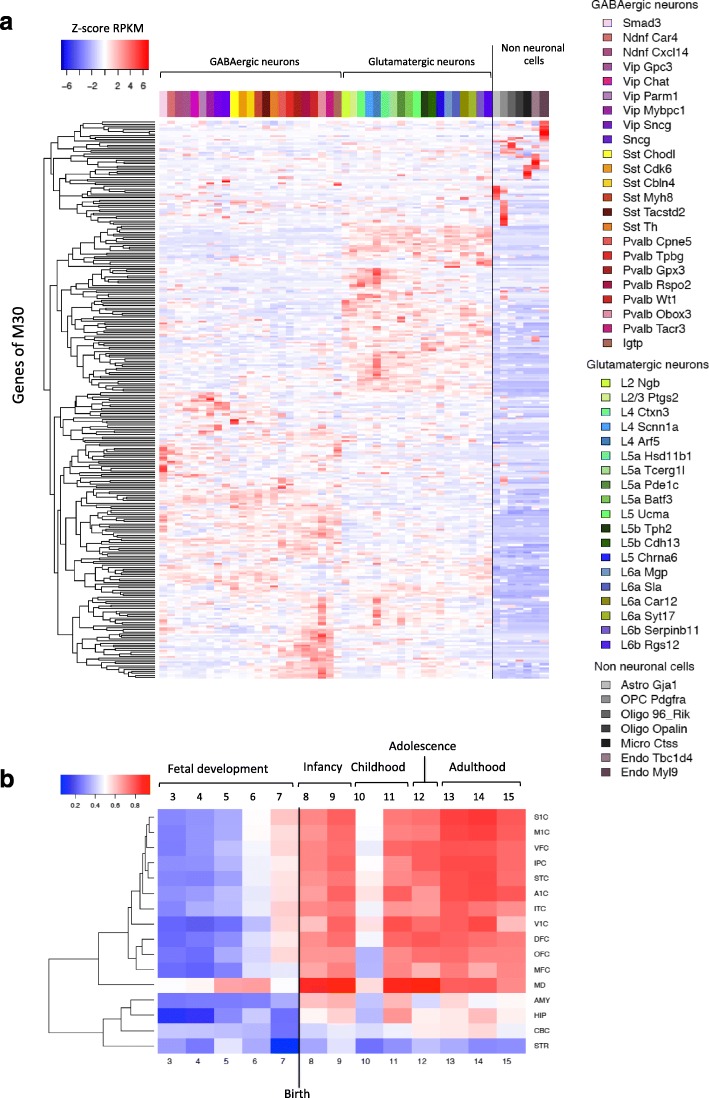
Expression of M30 genes across brain cell types and across whole life in distinct brain regions. **a** *Heatmap* of expression pattern of M30 genes across brain cell types and subtypes. *Colour bar* values represent scaled expression (SDs from the mean-centred expression value across cell-subtypes). **b** *Heatmap* of gradient of expression of M30 spanning fetal development to late adulthood and in topographically distinct brain regions. *A1C* auditory cortex, *AMY* amygdala, *CBC* cerebellar cortex, *DFC* dorsolateral prefrontal cortex, *HIP* hippocampus, *IPC* posterior inferior parietal cortex, *ITC* inferior temporal cortex, *M1C* primary motor cortex, *MD* mediodorsal nucleus of thalamus, *MFC* medial prefrontal cortex, *OFC* orbital prefrontal cortex, *S1C* primary somatosensory cortex, *STC* superior temporal cortex, *STR* striatum, *V1C* primary visual cortex, *VFC* ventrolateral prefrontal cortex

Since previous studies have shown that gene modules detected in the brain often correspond to (or reflect) expression in one or more cell types [38–40], we took advantage of recently published single-cell RNA-seq data [41] to annotate M30 for cell type expression (see ‘Methods’). Module M30 was significantly enriched in marker genes for interneurons (FET *P* value = 1.65 × 10^–8^, OR = 4.37) and pyramidal neurons (FET *P* value = 7.71 × 10^–4^, OR = 3.07). This mixed cell-type enrichment is consistent with the expression profile of M30 genes in the Allen Brain Institute single-cell RNA-seq brain dataset [42] (Fig. 3a). We then explored the expression of M30 genes in different stages of human brain development utilising data from Kang et al. [32] (see ‘Methods’) and observed a clear developmental gradient of expression of M30 genes beginning in early mid-fetal development (i.e. post-conception weeks 16–19), maximal by birth and then persisting through all post-natal periods (Fig. 3b). This clear developmental trajectory of expression of M30 led us to explore its transcriptional regulation. Using the WebGestalt toolkit [43] to test for enrichment of transcription factor binding sites (TFBS), we found M30 was highly enriched for NRSF/REST (repressor element 1-silencing transcription factor) targets (BH *P* = 2.55 × 10^–8^, *r* = 8.50). REST is a repressor of neuron-specific genes in early fetal development whose activity is downregulated in mature neurons [44] and dysregulated in a large array of brain pathologies including epilepsy [45–48]. The strong enrichment for REST-target genes in M30 is consistent with the tightly regulated developmental trajectory of expression of this module during brain development and supports the hypothesis that M30 genes non-randomly share a common regulation.

**Fig. 4:**
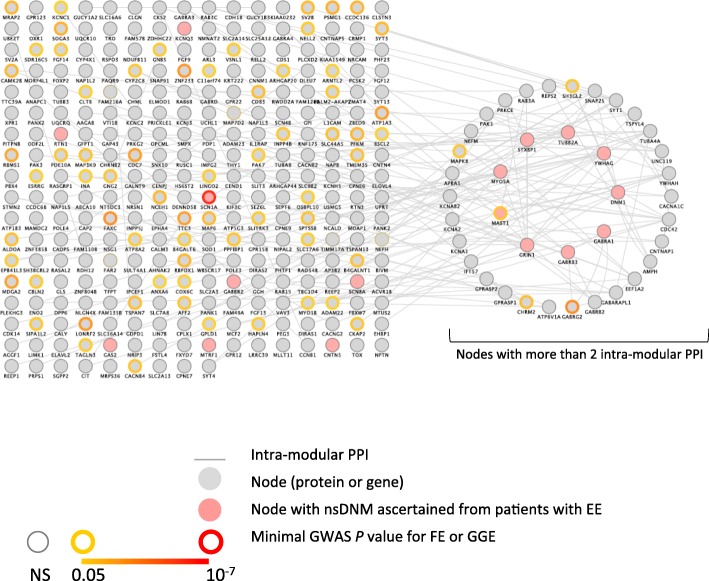
*Graphical representation* of the M30 co-expression and protein-protein interaction network and its relationship to epilepsy. Genes in M30 impacted by single nucleotide variant non-synonymous DNM from epileptic encephalopathy (EE) cases are filled in *light red*. The *bold border* of *circles* indicates genes showing nominal association with susceptibility by GWAS to generalised epilepsy (GGE) and/or to focal epilepsy (FE) using a *yellow* to *red* gradient colour according to the minimal *P* value. Genes disrupted by non-synonymous DNMs in EE patients are enriched in genes coding for proteins with higher number of intra-modular PPI (GSEA ranking genes according to the number of intra-modular PPI, NES: 2.4, FDR q-value: 0.0013; FET testing the enrichment of DNMs among genes coding for proteins with more than two intra-modular PPI: *P* value = 4.245 × 10^–4^, OR = 6.77)

We then investigated the physical interactions between the protein products of M30 genes. First, selecting sources of protein-protein interaction (PPI) from the STRING database [49], we found highly significant enrichment for PPI among M30 genes (expected number of PPI = 120, observed number of PPI = 217, enrichment *P* = 1.11 × 10^–15^) (see ‘Methods’). Then, we tested M30 for enrichment in PPI using the DAPPLE module [50, 51] of the GenePattern platform [52] and confirmed significant enrichment (expected Direct Edges Count (DEC): 59.33, observed DEC: 180, empirical *P* = 0.001). In addition, we collated non-redundant physical PPI data from three different sources: GeneMANIA [53], Hippie [54] and iRefWeb [55] (see ‘Methods’). Using experimentally derived physical interactions that could be mapped to gene names using HUGO Gene Nomenclature Committee database [56], our final database contained information for 272,348 non-redundant protein interactions for 17,235 genes, 13,489 of which are expressed in the UKBEC dataset. We then tested M30 genes for enrichment in proteins involved in intra-modular PPI compared to 100,000 simulated random control modules of similar size (see ‘Methods’). The M30 PPI network (Fig. 4) had significantly more nodes than the simulated PPI networks (142 nodes in the M30 PPI network out of 320 genes, empirical *P* = 0.02). This significant enrichment of the co-expression module M30 for direct PPI provides an independent line of evidence to support the validity of this module.

We then investigated the relationship between the topology of the M30 PPI network and epilepsy and to this aim we determined the degree of each node, i.e. the number of direct PPI/edges/connections that a node has with other nodes within the network. We found that genes disrupted by non-synonymous DNMs in monogenic EE patients are enriched in genes encoding proteins with a higher degree within the network (Gene Set Enrichment Analysis (GSEA) ranking genes according to the degree, Normalised Enrichment Score (NES) = 2.4, false discovery rate (FDR) = 0.1%). We also detected a relative enrichment of non-synonymous DNMs among genes coding for proteins with a degree superior to two (FET *P* value = 4.2 × 10^–4^, OR = 6.77) (see Additional file 3: Figure S3). This suggests that the genes in M30 impacted by non-synonymous DNMs in EE tend to be more highly connected within the PPI network (Fig. 4).

### Integration with common epilepsy variants

The genetic association of M30 with rare monogenic forms of epilepsy led us to explore the relationship between M30 and common forms of focal epilepsy (FE) and genetic generalised epilepsy (GGE), using GWAS data from the International League Against Epilepsy (ILAE) Consortium [15]. This meta-analysis of GWAS studies in common epilepsies consisted of 5310 FE cases and 23,606 controls and 2606 GGE cases and 18,990 controls. The enrichment of association of M30 to each phenotype was tested as previously described (see ‘Methods’) [25]. As a negative control, and to assess the specificity of the epilepsy GWAS enrichments, each module was also tested against five large GWAS of clinical phenotypes with no known relationship to epilepsy (waist/hip ratio, fasting insulin homeostasis, glucose challenge homeostasis, systolic blood pressure and diastolic blood pressure). We observed significant enrichment of association between M30 and focal epilepsy (FE: enrichment *P* = 0.00019, FDR < 5%, Z-score = 3.55) and between M30 and genetic generalised epilepsy (GGE: enrichment *P* = 0.0044, FDR < 5%, Z-score = 2.62) (see Table 1, Additional file 5: Table S4). None of the negative control GWAS datasets showed significant enrichment of association (see Additional file 5: Table S4). A constituent gene within M30 is *SCN1A*, which is a known genome-wide significant susceptibility gene for epilepsy [15, 54, 55]. We therefore re-tested the enrichment of association between M30 and epilepsy after excluding *SCN1A* from the list of M30 genes. After removing *SCN1A* from the set of M30 genes, the enrichment of association between M30 and epilepsy remained significant for both focal (FE: enrichment *P* = 0.0019, Z-score = 2.88) and generalised (GGE: enrichment *P* = 0.0097, Z-score = 2.34) epilepsy. In parallel, M30’s enrichment for non-synonymous rare variant DNM ascertained from patients with monogenic EE also remained highly significant after excluding *SCN1A* (FET *P* = 1.12 × 10^–7^, OR = 4.49, 95% CI = 2.52–8.03).

**Table 1:** Enrichment of association of M30 with epileptic and non-epileptic phenotypes

Genes^a^	*Z* score	*P* value**	Phenotype
278	2.62	0.00437	Genetic generalised epilepsy
297	3.55	0.00019	Focal epilepsy
277	2.34	0.00971	Genetic generalised epilepsy excluding *SCN1A*
296	2.88	0.00195	Focal epilepsy excluding *SCN1A*

**P value for enrichment of association

^a^Number of genes in the module with ≥1 genotyped SNP within the transcription start and end positions of the gene (GRCh37, hg19)

In summary, these integrative analyses suggest M30 as a convergent gene co-expression (and PPI) network involved in the genetic susceptibility to both rare monogenic and common polygenic forms of epilepsy. We therefore further explored M30 genes and investigated their relationship to epilepsy more broadly.

### Relationship of M30 expression to broad forms of epilepsy

We used genome-wide gene expression data from disease hippocampus tissues to investigate the association between expression of M30 genes and epilepsy. Differential expression analysis was carried out using gene expression data from: (1) the human epileptic hippocampus (24 TLE patients/23 controls) assayed by microarray; (2) the mouse epileptic hippocampus from the pilocarpine post-SE model of chronic TLE (100 cases/100 controls); and (3) the mouse hippocampus from a model of Dravet (*SCN1A*) disease (4 cases/ 4 controls) assayed by RNA-seq (see ‘Methods’). GSEA [57] was applied genome-wide to the ranked list of gene scores (reflecting both the significance and the magnitude of expression changes in epilepsy, see ‘Methods’) to test for enrichment of differentially expressed genes in the set of M30 network genes. The GSEA was carried out accounting for the direction of effect, i.e. considering whether a gene is upregulated or downregulated in each type of epilepsy.

M30 genes were significantly enriched for genes downregulated in the epileptic hippocampus in all three epilepsies (human TLE: NES = −4.51, *P* < 10^–5^; acquired post-SE TLE: NES = −5.83, *P* < 10^–5^; Dravet model: NES = −3.69, *P* < 10^–5^) (see Additional file 3: Figure S4). In contrast, the whole set of genes impacted by non-synonymous DNMs ascertained from EE patients was not significantly enriched for downregulated or upregulated genes (see Additional file 6: Table S5).

The observation that M30 is enriched for genes which are downregulated in multiple types of epilepsy led us to investigate the relationship between the level of expression of M30 genes and seizure frequency using the post-SE mouse model of TLE. Here, we used seizure frequency data obtained from 14 days of continuous video monitoring of epileptic mice beginning 28 days following pilocarpine induced SE [24, 58]. Using 1:1 orthologues, correlation of M30 gene expression levels with seizure frequency was undertaken using GSEA (see ‘Methods’). This analysis revealed a significant enrichment in M30 for genes whose expression in the epileptic hippocampus was anti-correlated to the frequency of seizures (NES = –6.68, *P* < 10^–4^) (i.e. increased expression of M30 genes correlates with fewer epileptic seizures, see Additional file 3: Figure S5). Taken together, these analyses suggest functional disruption by rare or common variation and/or downregulation of M30 gene expression as a convergent mechanism influencing susceptibility to epilepsy and seizures broadly. While the precise mechanistic connection between genetic disruption of M30 genes at the DNA sequence level and dysregulation of expression of M30 genes remains to be determined, the convergence of these orthogonal functional perturbations on M30 suggest targeting this network might represent a novel therapeutic opportunity.

### Valproic acid reverses the downregulation of M30

We hypothesised that M30 represents a potential candidate network to target as a therapeutic strategy. We therefore aimed to identify small molecules whose effect on gene expression could restore the downregulation of M30 genes observed in epilepsy toward health. One approach to identifying drugs with this property is to computationally ‘screen’ drugs according to the extent and specificity of the overlap between genes differentially expressed by a drug and the component genes in the disease network. To date, the largest database reporting empirical changes in cellular gene expression following drug exposure is Connectivity Map (CMap), which reports transcriptional signatures for 1300 compounds [28]. In order to screen drugs in CMap in terms of their predicted ability to restore M30 expression in epilepsy toward health, we first carried out differential expression analysis to identify genes with significant (FDR < 10%) drug-induced differential expression (DE) (see ‘Methods’). For the 152 drugs with ≥10 significantly differentially expressed genes in CMap, we tested the overlap of M30 genes with drug-induced DE, taking into account whether a gene was upregulated or downregulated by a given drug. Among the drugs whose induced transcriptional changes overlapped significantly with M30 in a therapeutic direction (see Additional file 7: Table S6), the strongest overlap was with genes differentially expressed by VPA (for MCF7 cell line with the concentration of 0.0005 mol/L, FET *P* = 9.93 × 10^–5^, BH *P* = 0.0089, OR = 4.81). Moreover, we observed a dose-dependent effect of VPA on M30 expression in a direction suggesting higher doses of VPA would have a greater therapeutic effect (see Additional file 3: Figure S6). VPA is an established and widely used AED with an established dose-dependent therapeutic response and a broad spectrum of clinical efficacy against different types of epilepsy (reviewed in [59]).

To seek replication for the predicted therapeutic effect of VPA on M30 gene expression, and to assess the relevance of gene expression changes observed in the cancer cell lines used by CMap to neurons, we analysed gene expression changes in 16 day neurons differentiated from mouse embryonic stem cells (mESCs) before and after treatment with VPA (here studying VPA as its sodium salt which is therapeutically equivalent to VPA) (see ‘Methods’). Using expression profiles from neurons treated with VPA and untreated control neurons representing the 5540 mouse genes with one-to-one human orthologues, and considering genes differentially expressed by VPA at FDR < 10%, we observed that VPA upregulates 51% of mapped M30 genes (significance of overlap compared to random expectation *P* = 4.9 × 10^–12^, OR = 4.53) and downregulates only 9% of mapped M30 genes (*P* = 0.013, OR = 0.42). These data confirm significant overall upregulation of M30 by VPA (see Additional file 3: Figure S7), replicating and strengthening the results obtained from the analysis of VPA-induced DE in the cancer cell lines from CMAP. While these in vitro results will ultimately require validation in vivo, they provide replicable evidence for the potential to use drug-induced upregulation of the epilepsy-related M30 network as a potential novel therapeutic strategy.

## Discussion

The systematic integration of diverse genomic datasets, including brain gene expression data, de novo mutations for rare monogenic epilepsies and GWAS data from common epilepsy reported here, identified a convergence of genetic susceptibility for epilepsy on a gene-regulatory network (M30). The M30 network consists of 320 genes co-expressed across the human brain under tight developmental control. We observed that rare de novo mutations for monogenic epilepsy preferentially impact the mostly highly connected genes in the network. These results provide evidence for a shared pathogenesis between the rare and polygenic forms of epilepsy, although whether the enrichment of association of M30 with polygenic epilepsy reflects tagged rare variant contributions to the disease or true enrichment of common variants remains to be determined.

The absence of significant enrichment in non-synonymous DNMs ascertained from patients with ID, ASD or SCZ for any brain co-expression network was unexpected given the emerging evidence for overlap in genetic susceptibility between the different brain developmental disorders [60] and the previously demonstrated higher connectivity among genes mutated in SCZ [61] and ASD [62]. One potential biological explanation is that the pathways disrupted in these diseases (in contrast to EE) are specific to a particular stage of neurodevelopment and so unrepresented in the co-expression networks we built from adult human brain samples [61, 62]. Alternatively, the heterogeneity of pathways disrupted in EE may be less than for some other neuro-developmental diseases or potentially, the disrupted pathways are specific to a particular cell type which is not captured by co-expression analysis in bulk brain tissue. Future analyses using cell-type specific RNA-seq at different stages of development will more fully resolve the co-expression relationships between genes for the different neurodevelopmental diseases.

Functionally, the M30 module is highly enriched for genes involved in neural processes and synaptic function. The network is highly expressed in interneurons and pyramidal neurons, is enriched for genes that are downregulated in the epileptic hippocampus in heterogeneous forms of epilepsy including human TLE, a mouse model of acquired TLE and a mouse model of monogenic Dravet (*SCN1A*) disease, and M30’s expression is significantly negatively correlated with seizures. These results suggest that M30 may play a role in maintaining the homeostatic balance between excitation and inhibition in the mammalian brain which, when disrupted by either genetic variation or altered expression, contributes to epilepsy susceptibility and seizures. These findings suggested targeting the expression of M30 as a potential new therapeutic strategy.

At the level of protein-protein interactions, several studies based on whole-exome sequencing trio analysis of neurodevelopmental disease have reported that genes with de novo loss-of-function mutations code for highly interconnected protein networks for autism [63], schizophrenia [61] and EE [14]. In keeping with this, we found that the protein products of M30 genes are also significantly enriched for direct protein-protein interactions. In protein networks, the nodes with high degree (i.e. highly connected proteins), also termed hubs, are often functionally important. The most striking examples of this were initially discovered in worm, fly, yeast and *E. coli* proteomes, leading to the ‘centrality-lethality’ hypothesis which proposes that nodes with higher centrality in a network are more likely to produce disease phenotypes when removed compared to nodes with lower centrality [64–66]. In humans, the location of genes that cause disease within a network is more debated. Defining human essential genes as orthologues of mouse genes that result in embryonic or postnatal lethality when disrupted by homologous recombination, it was argued that it is the essential genes, and not the disease genes, that encode hubs [21, 67]. However, the centrality hypothesis of disease genes has been put forward several times, particularly in cancer [68], but also more generally for genes with phenotype-causing mutation in OMIM [69]. A more recent study on the structural controllability of human directed protein networks defined indispensable proteins and demonstrated that these proteins are enriched in disease-causing mutations [70]. Whether the centrality of genes disrupted by non-synonymous DNM in highly penetrant (often monogenic and severe) forms of epilepsy will hold true also for genes harbouring common risk variants identified from large-scale epilepsy GWAS remains to be determined.

The analysis of differential expression of M30 genes in epileptic brain tissue versus controls showed a consistent enrichment in the M30 module for downregulated genes in epilepsy. Using quantitative data of seizure frequency in epileptic mice, we observed a significant enrichment in M30 for genes whose expression in the epileptic hippocampus was anti-correlated to the frequency of seizures (higher M30 expression correlated with fewer seizures). These analyses, together with genetic association of M30 to both monogenic and polygenic forms of epilepsy, suggest M30 may play a role in the mechanistic pathways leading to epilepsy. This led us to hypothesise that targeted increases in M30 expression could be exploited as a novel therapeutic approach in epilepsy.

Using the CMap dataset, we prioritised several drugs and in particular VPA, a widely used AED, as having a preferential and therapeutic effect on M30 expression. No other established AED had a significant effect on M30 gene expression in the CMap database and the identification of VPA as a candidate therapy for epilepsy was entirely independent of its known role in epilepsy or mechanism of action [59, 71] and based solely on an unsupervised analysis of disease and drug-related differential gene expression. Although VPA has a rapid onset of anticonvulsant activity, suggesting a direct effect on neuronal electrophysiology, the anticonvulsant activity of VPA has also been noted to increase during prolonged treatment [72], suggesting a potential transcriptional mechanism mediating its antiepileptic activity. These unsupervised analyses therefore suggest a systematic screen of drug-like molecules based on their measured effect on M30 gene expression might represent an efficient therapeutic strategy for new drug discovery in epilepsy. In keeping with this, of the 15 drugs in CMap predicted to result in an overall significant upregulation of M30 (see Additional file 7: Table S6), whitaferin A (WFA) is a steroidal lactone present in *Withania somnifera* (also known as Indian ginseng or Ashwagandha), a plant that has been used in Ayurvedic medicine for centuries in the treatment of a wide range of diseases including epilepsy [73] and studies in animal models have also reported the anticonvulsive effect of this drug [74, 75].

Since M30 is highly expressed in interneurons and pyramidal neurons our analyses do not currently distinguish between specific downregulation of M30 in neurons or secondary neuronal loss in epilepsy. Loss of neurons and interneurons can be a feature of human TLE [76], as well as some animal models of acquired epilepsy [77]. However, in Dravet disease, despite evidence for interneuron dysfunction [78], neuropathological analyses have shown a striking preservation of interneurons and neurons despite decades of poorly controlled epilepsy [79]. Consistent with this, our analysis of changes in expression of cell type marker genes (see Additional file 6: Table S5) found no evidence for interneuron loss in the Dravet, mouse model, although did suggest pyramidal neurons may be lost in Dravet. Future analyses focusing on single cell-type sequencing in the epileptic brain will be required to distinguish between downregulation of M30 specifically or differential neuron/interneuron loss. However, regardless of the mechanism of reduced M30 expression in epilepsy (i.e. specific downregulation in epileptic neurons/interneurons or differential cell loss), the suggestion of targeting upregulation of M30 network as a means of compensating for the resulting functional deficit is unaltered as a potential therapeutic rationale.

## Conclusions

In conclusion, our results implicate a convergent role for a synaptic gene network (M30) in rare monogenic and common (presumed polygenic) forms of epilepsy and suggest targeting the expression of this network is potential new therapeutic strategy in epilepsy. Further studies will be required to clarify the specific contribution of M30 genes to ictogenesis and to identify gene-regulatory factors or drugs that influence M30 expression in a therapeutic direction.

## Methods

### Weighted gene co-expression network analysis of nine brain regions post-mortem human samples

#### Brain region expression datasets

The UK Brain Expression Consortium (UKBEC) released genome-wide gene expression data from several brain regions of individuals of European descent (GSE60862) [80]. We selected samples for which data were available for the same 88 neuropathologically normal individuals in all the nine following brain regions: cerebellar cortex, frontal cortex, occipital cortex, temporal cortex, hippocampus, putamen, thalamus, medulla and white matter.

Raw expression profiles from the Affymetrix Human Exon 1.0 ST Array were processed to transcript-level expression with Affymetrix Power Tools (APT) (http://www.affymetrix.com/partners_programs/programs/developer/tools/powertools.affx) using probe logarithmic intensity error (PLIER) normalisation [81] with probe GC-content correction. Only the most reliable ‘core’ set of probes was used to generate transcript level expression profiles as defined in the Affymetrix chip definition file. Probes were retained if more than 50% of the samples in at least one brain region that had detection background *P* values < 0.01. Gene-level expression was obtained by taking the median of the expression values of multiple exons mapping to the same gene. These filtering steps defined a final dataset of 20,388 probes, representing 15,199 protein coding unique genes (hg19/GRCh37/Ensembl version 75), which were then used after quantile normalisation for network analysis and as the ‘background’ gene set for enrichment analyses.

Before inferring gene co-expression networks, we used the probabilistic estimation of expression residuals (PEER) package [82] to determine hidden factors that explain much of the expression variability in the dataset. We used principal component analysis (PCA) to calculate summary variables describing the variation in the microarray expression of the 15,199 genes and estimate the potential effects of the hidden factors identified by PEER and of the known clinical covariates on global gene expression variability. The plotting of hidden factors across samples allowed us to select the hidden factors F1 and F4 as potential batch effect without high relation to the variability explained by brain regions (see Additional file 3: Figure S1). Gene expression levels were therefore adjusted to remove the effect of the selected hidden factors and covariates (F1, F4, age, gender, post-mortem interval, cause of death and brain-bank ID) by fitting linear models on gene expression using the *lm* function in R. The residuals from the linear model were then used as expression values (for 15,199 genes in 88 samples of each brain region dataset).

#### Co-expression network construction

Genes were then grouped into modules using co-expression network approaches on the nine brain regions datasets: consensus method implemented in WGCNA to pinpoint co-expression modules common to the nine brain regions and DiffCoEx [30] to construct modules of differentially co-expressed genes in one or several regions compared to the others.

The consensus co-expression network analysis was carried out using the *blockwiseConsensusModules* function in the WGCNA R package as previously described [83, 84], with the following parameters: β = 7 (chosen based on the scale free topology criterion r^2^ > 0.8), minModuleSize = 40, mergeCutHeight = 0.25, minBlockSize = 20,000, corType = ‘bicor’ (to compute robust pairwise correlations of gene expression using Tukey’s biweight method [85]). Briefly, for each brain region a pairwise correlation matrix was calculated and then converted to an adjacency matrix by raising it to the β power. For each pair of genes, topological overlap matrix (TOM) was calculated based on the adjacency matrix. After the TOM of each brain region was scaled to make them comparable, the consensus TOM was calculated by taking the component-wise minimum of the TOMs of each individual brain region. The consensus TOM was then clustered using average hierarchical clustering based on the dissimilarity of gene connectivity, defined as 1 – consensus TOM. Consensus co-expression modules were defined as branches of the resulting clustering tree, using dynamic tree-cutting [86].

The differential co-expression network analysis was carried out using the DiffCoEx method with its variant for multiple conditions [30]. DiffCoEx analyses were carried out in R following the procedure described [30]. Briefly, DiffCoEx builds on WGCNA framework by computing a TOM generated from a matrix of adjacency differences between conditions, therefore it focuses on detecting the differences in co-expression patterns between these conditions. Tukey’s biweight method [85] was used to compute robust pairwise correlations of gene expression and applied β = 7 as soft thresholding value.

### Testing preservation and reproducibility of the UKBEC consensus and differential modules in other datasets

Several independent brain gene-expression datasets were used to establish that the modules derived from the UKBEC samples were reproducible in the healthy human brain during development and at different ages across life, in different brain regions and tissues, and in the hippocampus of mice (see Additional file 2: Table S2).

To test spatio-temporal preservation of modules, we used transcriptome data from GSE25219 [32, 33]. These microarray expression data were generated using Affymetrix Human Exon 1.0 ST array analysis of 16 brain regions from 1263 samples collected from 53 clinically unremarkable post-mortem human brains, spanning embryonic development to late adulthood. The quantile normalisation and filtering using expectation maximisation (EM) algorithm was done as previously described [25]. The RNA-seq expression data from the GTEx project was used to analyse the preservation of co-expression modules across different brain regions but also in other tissues. The fully processed, normalised and filtered gene level expression data were downloaded from http://www.gtexportal.org/(GTEx_Analysis_V6). The raw data of the E-MTAB-3123 dataset of epileptic and normal human hippocampi were downloaded from https://www.ebi.ac.uk/arrayexpress/experiments/E-MTAB-3123/. Data normalisation, adjustment and filtering were applied as previously described [87]. We also used the human post-mortem hippocampus microarray expression data from 63 healthy post-mortem human brains publicly available from Pritzker Neuropsychiatric Disorders Research Consortium (http://www.pritzkerneuropsych.org/?page_id=1196, GSE45642). These data were processed as previously described [25]. Finally, to assess whether the modules were preserved in another species we used the RNA-seq data from mouse hippocampi previously generated and processed [24]. The datasets were annotated with human Ensembl gene ID using the biomaRt Bioconductor R package [88, 89] and selecting human genes that were ‘one-to-one’ orthologues with mouse genes.

We used the function *modulePreservation* of WGCNA R package to calculate the Z_*summary*_ value for each module [31]. This function computes a permutation test to estimate the mean and variance of several preservation statistics under the null hypothesis of no relationship between the module assignments in the reference and test data. The Z_*summary*_ value summarises density and connectivity based Z statistics generated through these permutations and therefore is indicative of the module robustness and reproducibility. In general, modules with Z_*summary*_ scores above 10 are interpreted as strongly preserved (that is, densely connected, distinct and reproducible modules), Z_*summary*_ scores between 2 and 10 are weak to moderately preserved and Z_*summary*_ scores below 2 are not preserved.

### Assessing enrichment in rare de novo mutations in neurodevelopmental disorder

DNM reported in published neurodevelopmental trio WES studies were collated: EE (*n* = 356) [13, 14], ASD (*n* = 4186) [90, 91], SCZ (*n* = 1004) [61, 92–95] ID (*n* = 192) [96–98] and DDD (*n* = 1133) [99, 100]. For controls, we used 1891 non-neurological control samples from seven published studies [61, 63, 90, 94, 96, 101, 102].

To integrate these data though their variable sources and coverage, we assumed each gene has 100% of its CDDS sequence covered across all trios, as previously described [25]. For each disorder, we included SNV DNM considering all missense, nonsense and splice-site SNV mutations. We adopted a FET (two-tail) to empirically compare the rates of DNMs overlapping the CCDS real estate of a module in case- and control cohorts. The code of the in-house R function to compute enrichment in DNMs is available at https://github.com/adelahay/BrainCell (*DNMFET* function).

### Protein-protein interaction network analysis

First, to test the enrichment of PPI among protein products of M30 genes, we used the tool provided in the version 10.0 of STRING database [49]. The enrichment was calculated selecting known interactions (experimentally determined and from curated databases). Second, we tested M30 for enrichment in PPI using the DAPPLE module [50, 51] of the GenePattern platform [52]. DAPPLE looks for significant physical connectivity among proteins encoded for by genes according to protein-protein interactions reported in the InWeb database [103]. Then, we collated protein-protein interactions (PPI) from three different databases: GeneMANIA [53], Hippie [54] and iRefWeb [55]. We created 100,000 random gene sets that each contained 320 different genes expressed in the UKBEC dataset (320 corresponding to the number of genes of M30). PPI networks were constructed for M30 module and for each gene set and we used these simulated networks to generate normal distributions of PPI networks. The *P* value for enrichment of genes involved in PPI was estimated by the proportion of control networks with more genes involved in PPI network than the 142 ones of M30.

### GWAS-enrichment analysis

To test for enrichment of genetic association in a gene-set (i.e. co-expression module) we used versatile gene-based association study (VEGAS2) [104] to generate a gene-based association statistic (*P* value) controlled for the number of SNPs in each gene and the LD between those SNPs. In all analyses gene-based *P* values were calculated using VEGAS2 and the top 10% option with 100,000 iterations and a gene window consisting of the transcriptional start and stop position of each gene. For the ILAE Consortium on Complex Epilepsies and the control GWAS datasets of waist-hip ratio, fasting insulin homeostasis, glucose challenge homeostasis, systolic blood pressure and diastolic blood pressure, the default 1000 Genomes European population was used to control for LD in the VEGAS2 analysis. The GWAS-enrichment statistic was calculated for the tested modules from the gene-based association *P* values (from VEGAS2) using the Z-test based bootstrapping method [105] (one-sided) where, for each network, 100,000 random gene sets of same size as the network were sampled from the list of all brain regions expressed genes (*n* = 15,199). We tested enrichment M30 module for association to several disease traits and used FDR method to account for the number of tests.

### Enrichment in differential expressed genes between cases and controls in epileptic patients and mice models of epilepsy

Differential expression analysis was performed in three datasets (see Additional file 2: Table S2). For human microarray hippocampus expression dataset E-MTAB-3123 (24 TLE patients/23 controls, https://www.ebi.ac.uk/arrayexpress/experiments/E-MTAB-3123/[90]), the differential expression analysis of the adjusted dataset was performed using the limma package [106]. The mouse datasets were annotated with human Ensembl gene ID using the biomaRt Bioconductor R package [88, 89] and selecting human genes that were ‘one-to-one’ orthologues with mouse genes. The package EdgeR [107] was used for differential analysis of RNA-seq mouse hippocampus datasets for both models, the pilocarpine mouse model (100 cases/100 controls) and the Dravet syndrome mouse model (4 cases/ 4 controls). The pilocarpine mouse model is a mouse model of temporal lobe epilepsy (TLE), where the mice develop spontaneous recurrent seizures (SRS) (i.e. epilepsy) a few weeks following the induction of status epilepticus (SE) by an injection of pilocarpine [24, 58, 108]. The Dravet syndrome mouse model is a *Scn1a* knock-in mouse model (Mouse Genome Informatics MGI:3713740) for which the hippocampi were collected at 14 days of age, prior to the onset of seizures [109, 110]. For the Dravet syndrome mouse model experiments, only male mice were selected and healthy littermate mice were used as controls.

We multiplied the adjusted *P* value (the –log10 of *P* value) by the log-transformed fold change to generate a gene-level score, which was used as a metric to ‘rank’ genes. This gene-level score reflects the significance and the intensity of differential expression. Gene set enrichment analysis (GSEA) [57] was then used to test if a group of genes (genes from respective co-expression module) occupy higher (or lower) positions in the ranked gene list than what it would be expected by chance. Gene set enrichment scores and significance level of the enrichment (NES, *P* value, FDR) and enrichment plots were provided in the GSEA output format developed by Broad Institute of MIT and Harvard (permutations = 100,000).

### Enrichment in transcripts anti-correlated with frequency of seizure in the pilocarpine mouse model

To this aim, we used data obtained from 14 days of continuous video monitoring of behavioural seizures in the mouse model of TLE beginning 28 days following pilocarpine induced SE) [24, 58]. For all genes, the Spearman correlation coefficient between the number of seizures and the level of its expression was calculated using the *cor.test* function in the stat R-package. We multiplied the –log10 of *P* values by the correlation coefficients of each Spearman’s test to generate a gene-level score, which was used as a metric as described above to ‘rank’ genes. GSEA [57] was applied to the genome-wide ranked list of gene scores to test for enrichment of correlated genes or anti-correlated genes in the set of M30 genes.

### Spatiotemporal analysis of module expression

To determine the spatiotemporal expression dynamics of modules, we used quantile normalised gene level expression values (log_2_ transformed) from GSE25219 [32]. These transcriptome data were generated using Affymetrix Human Exon 1.0 ST array analysis of 16 brain regions comprising the cerebellar cortex, mediodorsal nucleus of the thalamus, striatum, amygdala, hippocampus and 11 areas of the neocortex. The data were generated from 1263 samples collected from 53 clinically unremarkable post-mortem human brains, spanning embryonic development to late adulthood (from 10 weeks of post-conception to 82 years of age, which corresponded to periods 3–15, as previously designated) [32]. The log_2_-transformed gene expression data follow a bimodal distribution contributed by low (likely non-functional) and high expressed genes [111]. We used the expectation maximisation (EM) algorithm to model gene expression levels as a mixture of normal distributions and identify the underlying distributions of low and high expressed genes. Only the genes, with mean of log_2_-transformed expression values over the 95 % percentile of distribution of low-expressed genes (here > 5.61) were considered for further analysis (*n* = 8704). The EM algorithm was implemented using *normalMixEM* function from the mixtools R package. Spatio-temporal dynamics of co-expression modules M1 and M3 across 16 brain regions and 13 developmental time points were illustrated as a heatmap, as previously described [33]. Module expression for each region and developmental time point was calculated by averaging the scaled expression across all genes in a module. The resultant heatmap graphs illustrate the changes in expression of genes of a co-expression module across brain development and cortical regions. The code of the in-house R function to build spatiotemporal heatmap is available at https://github.com/adelahay/BrainCell (*BrainH* function).

### Cell-type analysis

A publicly available set of cell-type marker genes was used to determine whether the network is enriched for genes specific to a particular cell type [41]. Marker genes were defined as preferentially expressed in a particular cell type, using single cell RNA-seq from mouse cortex and hippocampus for nine cell types [41]. All expressed background genes used to build these sets of marker genes were annotated with human ENSG gene ID using the biomaRt Bioconductor R package [88, 89] selecting ‘one-to-one’ orthologues. Module enrichment for each cell type was performed using FET to empirically compare the rate of genes overlapping the cell-type marker genes within and outside the module. The code of the in-house R function to compute enrichment in cell-type marker genes is available at https://github.com/adelahay/BrainCell (*CellFET* function).

To evaluate the pattern of expression of the M30 genes across cell types, we also used another single-cell RNA-seq mouse brain dataset [42]. The processed and normalised gene level expression values (reads per kilobase par million mapped reads (RPKM)) from GSE71585 [42]. We selected the 1424 single-cell data that were classified consistently into one of the 49 cell types (core cells). To display the changes of expression across cell types, the expression in one cell type was calculated by averaging the expression of all cells of this cell type. The M30 genes average expression values were then scaled and plotted as a heatmap to show the change of gradients across cell types. The code of the in-house R function to build the cell-type heatmap is available at https://github.com/adelahay/BrainCell (*CellTax* function).

### Drug repurposing analysis

We analysed Connectivity Map (CMap, build 02, http://www.broadinstitute.org/cmap/) dataset that collected from four human cell lines treated with ~1300 drugs, including many Food and Drug Administration (FDA) approved, in different concentrations. Here, genome-wide expression profiling was conducted after 6 h using the Human Affymetrix U133 microarray platform. Raw microarray data from CMap were downloaded and processed using systematic analysis of the impact of custom (CDF) on GeneChip data analysis [112, 113], RMA method as implemented in the ‘affy’ R-package [114], and were normalised using variance stabilisation with the ‘vsn’ R-package [115]. We selected drugs for which data were available in at least one replicate and performed differential expression analysis between expression profile in cells with the drug and control cells using the limma package [106]. After filtering of significant differentially expressed genes with a cutoff at 10 % FDR, we obtained lists of significantly upregulated and/or downregulated genes for 152 drugs in at least one of the three major cell lines of CMap (HL60, human promyelocytic leukaemia cell line; MCF7, human breast adenocarcinoma cell line; PC3, human prostate cancer cell line). For each drug, we tested the overlap of M30 genes with the list of genes upregulated by that drug using one-tail FET (in order to prioritise drugs predicted to reverse the downregulation of M30 genes observed in epileptic hippocampi). We then used the BH method to correct the *P* values for multiple hypotheses testing [116].

### Replication of enrichment in valproic acid induced upregulated genes

To replicate the predicted significant upregulation of M30 by VPA using CMap, we utilised gene expression data in neurons (Gene Expression Omnibus accession GSE50215). Here, genome-wide gene expression data were generated on day 16 neurons differentiated from mESC before and after treatment with sodium valproate (the sodium salt of VPA) using Affymetrix Mouse Gene 1.0 ST Array. Gene expression data were obtained from three biological replicates of vehicle (PBS) and VPA-treated cells. Here again, raw microarray data were processed using custom CDF [112, 113, 117], the RMA method as provided in the ‘affy’ R-package [114] and normalised using variance stabilisation with the ‘vsn’ R-package [115], differential expression analysis using the limma package [106]. Mouse genes were mapped to human one-to-one orthologues (5540 Ensemble gene names). The significance of the overlap between genes upregulated by VPA and genes in M30 genes was calculated as above.

## Additional files

Additional file 1: Top functional enrichment in GO biological process for gene co-expression modules inferred from UKBEC nine brain regions using consensus WGCNA and DiffCoEx. In second and third sheet: gene lists for the gene co-expression modules. (XLSX 2435 kb)
Additional file 2: Expression datasets used in this study. (XLSX 35 kb)
Additional file 3: All additional figures. (PDF 661 kb)
Additional file 4: Enrichment of non-synonymous DNM from patients with neurodevelopmental disease. (XLSX 107 kb)
Additional file 5: Enrichment of association of M30 with epileptic and non-epileptic phenotypes. (XLSX 34 kb)
Additional file 6: Enrichment for differentially expressed genes in epileptic hippocampi versus controls for several sets of genes. (XLSX 57 kb)
Additional file 7: Overlap between M30 and significantly upregulated genes after 6 h of treatment. (XLSX 37 kb)
