# Supplementary material for: Rare and common epilepsies converge on a shared gene regulatory network providing opportunities for novel antiepileptic drug discovery
Source: Genome Biol. 2016 Dec 13;17:245. doi: 10.1186/s13059-016-1097-7 (PMC5154105; doi:10.1186/s13059-016-1097-7)

Supplementary Figures

Supplementary Figure 1: Plots of the values for each sample for the 6 first hidden factors as defined using PEER. 2
Supplementary Figure 2: Heatmap of module preservation scores for modules identified in UKBEC in other expression datasets, across different brain regions, across pre-natal and post-natal life, in other non brain tissues, and in mouse hippocampus. 3
Supplementary Figure 3: Gene Set Enrichment Analysis (GSEA) plot of non synonymous *de novo* mutations (nsDNM) in patients ascertained for epileptic encephalopathy (EE) for genes coding for proteins with higher degree in the M30 protein-protein interaction (PPI) network. 4
Supplementary Figure 4: Differential expression analysis of M30 genes in epileptic hippocampus in three epilepsies. 5
Supplementary Figure 5: GSEA plot of M30 for genes anti-correlated to seizure frequency in the pilocarpine mouse model of chronic TLE. 6
Supplementary Figure 6: Dose effect on significance of enrichment of M30 for genes significantly upregulated by valproic acid. 7
Supplementary Figure 7: Mapped M30 genes which are up or down regulated by valproic acid. 8

Supplementary Figure 1: Plots of the values for each sample for the 6 first hidden factors as defined using PEER.

The circles are colored depending on the brain region of the sample. The hidden factors F1 and F4 were selected, as their values didn't match the variability possibly due to brain regions.

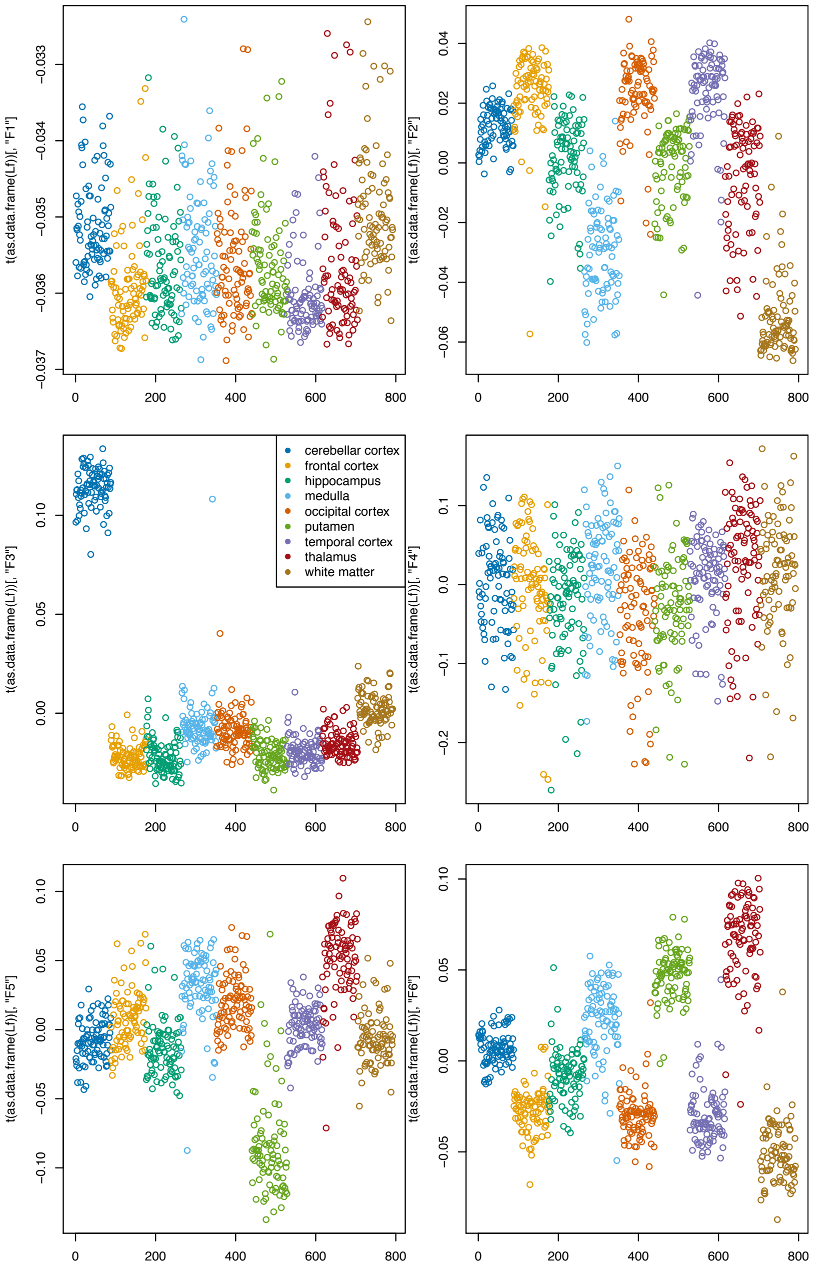

Supplementary Figure 2: Heatmap of module preservation scores for modules identified in UKBEC in other expression datasets, across different brain regions, across pre-natal and post-natal life, in other non brain tissues, and in mouse hippocampus.

Module conservation was assessed using the $Z_{summary}$ value [31], which quantifies the degree of module preservation between gene expression datasets. Moderate preservation of co-expression is indicated by a $z_{summary}$ score of >5) and strong preservation by a $Z_{summary}$ score >10.

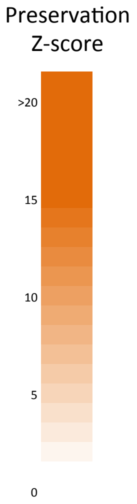

Brainspan microarray

Group	Module	foetal_AMY	foetal_CBC	foetal_HIP	foetal_MD	foetal_STR	foetal_neoCTX	mature_AMY	post-natal_CBC	post-natal_HIP	post-natal_MD	post-natal_STR	post-natal_neoCTX
consensus	M0	1.6	6.9	1.7	1.9	4.1	3.1	3	3.5	8.3	7.7	9.5	6
	M1	7.9	7.9	6	5.4	7.8	4	29	8.3	27	24	16	26
	M2	16	12	11	20	27	7.5	25	22	25	48	19	58
	M3	17	2.8	17	6.3	12	14	15	3.8	20	9.4	19	18
	M4	5.8	0.7	5.9	1.6	3.8	4.6	12	5.8	18	4.7	18	14
	M5	5.1	4.1	8.6	9.7	7.7	6.8	12	14	9.1	13	8.9	14
	M6	4.8	5.7	3	4.8	5.9	3.8	14	4.2	13	10	12	11
	M7	1.8	0.2	1.3	0.82	3.6	-0.02	12	1.7	12	11	11	13
	M8	3.4	4	3.2	7.8	8.3	3	5.8	5.8	8.1	11	2.8	14
	M9	4.3	3.1	3.5	2.4	3.3	4.9	6.4	4.7	9.5	5.8	6.6	8.6
	M10	6.9	10	11	7.3	9.5	7.3	11	14	8.2	13	16	14
	M11	2.5	2.9	2.6	1.1	3.6	1.3	5.7	2.2	2.4	1.6	3.9	3.7
	M12	11	7.9	5.6	8.9	7.8	8	11	7.8	16	5.8	9.8	12
	M13	3.4	1.7	2.7	0.48	4.1	1.3	4.5	1.5	4	2.8	3.1	3.9
	M14	3.8	3.1	7.7	3.6	6.4	2	13	11	10	19	7.1	16
	M15	17	14	8.8	9.1	13	11	19	24	19	13	16	19
	M16	6.9	6.8	3.9	5.4	4.9	4.2	11	11	9.4	12	11	14
	M17	0.3	0.8	-0.02	0.27	0.38	0.23	0.14	1	0.07	0.5	0.05	1.1
	M18	5.7	0.83	5.3	4	6.4	3.3	3.3	1.6	7.5	8.6	4.2	11
	M19	4.3	3.3	2.7	4.1	5.8	3.1	7.1	11	6.9	11	8.7	11
	M20	5.4	4.1	4	4.8	6.3	4.2	11	8.2	9.2	8.1	9.7	8.6
	M21	3.7	3.9	4.2	2.5	6.6	3.1	12	2.9	11	9	8	9.1
	M22	2.2	0.23	2.3	2.6	3.1	3	3.7	2.5	4.3	4.1	5.6	4.1
	M23	10	9.4	6.9	7.5	11	7.4	30	10	25	20	17	26
	M24	1.8	4.1	-0.01	1.5	2.7	-0.67	33	8.5	44	17	50	49
	M25	6.1	2.7	4.8	5.1	9	4.7	13	5.8	13	11	13	14
	M26	5.3	1.9	5.5	1.5	4.8	5.5	7.5	0.83	9.7	2.8	5	9.2
	M27	2.9	0.89	0.96	1.2	2.5	2	4.5	3.4	4.1	2.2	2.8	2
	M28	3.9	2.5	1.2	5.7	2.2	1.2	17	4.9	17	14	10	14
	M29	4.3	4.5	4.3	3.7	4.9	3.3	7.6	2.8	6.9	4	7.4	7.8
	M30	13	4.7	14	4	11	6.9	20	9.9	22	38	9.5	20
	M31	8.8	8.8	5	3.4	6.2	3.4	21	6.6	19	17	14	17
	M32	2.4	4.1	2	3.4	4.5	2.9	3.5	12	4.1	3.3	6.5	6.1
	M33	5.1	5.5	5.6	2.5	8.1	2.7	14	3.7	12	16	9.1	11
	M34	2.5	0.52	2.1	0.93	2.3	1.7	4.6	2.5	7.5	2.1	8.4	4.8
DiffCoEx	M0	2.3	2.8	5	1.2	1.7	3.7	5.2	4.4	6.9	1	6.2	3.3
	M1	8.7	3.8	7.6	6.1	10	7.4	16	7.8	25	8	18	17
	M2	18	15	15	26	33	8.6	33	25	32	60	23	61
	M3	1.8	3.6	3.6	0.76	1.6	1.4	1.2	1	12	4.1	0.47	4
	M4	4.8	1.7	3.3	4.4	4.2	2.2	12	5.9	9.5	6.8	9.2	9.1
	M5	1.8	1.4	1.1	1.2	2	1.4	5.7	2.9	4.9	22	3.7	7.3
	M6	1.8	3.7	0.45	4.3	3.9	1	33	8.1	35	16	37	38
	M7	2.6	9.1	3	3.7	9.2	2.4	14	2	17	19	7.9	12
	M8	1.3	1.8	0.26	1.3	4.1	0.49	0.03	5	4	0.53	2.8	2.6
	M9	5.7	7.2	10	3.9	9.7	3.2	21	11	20	26	13	21
	M10	7.4	4.8	7	6.3	10	5.6	14	10	12	11	15	15
	M11	12	8.9	9.8	12	9.5	9.3	24	14	26	12	20	27
	M12	14	9.9	17	3.9	10	9.9	24	3.5	27	26	6.4	24
	M13	14	8.6	6.6	12	9.9	9.9	11	9.5	18	6.3	11	14

GTEX RNAseq / Mouse

Group	Module	nGTEX_Anterior_cingulate_cortex_BA24	nGTEX_Caudate_basal_ganglia	nGTEX_Cerebellar_Hemisphere	nGTEX_Cerebellum	nGTEX_Cortex	nGTEX_Frontal_Cortex_BA9	nGTEX_Hippocampus	nGTEX_Hypothalamus	nGTEX_Nucleus_accumbens_basal_ganglia	nGTEX_Putamen_basal_ganglia	nGTEX_Whole_Blood	nGTEX_Cells_EBV-transformed_lymphocytes	nGTEX_Cells_Transformed_fibroblasts	nGTEX_Skin_Not_Sun_Exposed_Suprapubic	nGTEX_Nerve_Tibial	nGTEX_Muscle_Skeletal	Pritzker_hippocampus_controls	EMTAB3123_hippocampus_controls	UCB_hippocampus_controls
consensus	M0	12	7.1	2.9	4.6	15	14	16	9.1	9.4	12	1.3	3.8	2.8	3.6	4.6	2.6	4.4	7.4	3.3
	M1	28	22	26	26	25	26	21	31	29	19	10	6	5.7	16	17	12	6.1	21	16
	M2	43	34	36	44	44	46	33	23	36	34	0.23	-1.2	1	8.4	6.4	4.1	30	40	5.6
	M3	20	31	4.1	9.3	27	23	40	25	29	28	3.3	-0.01	0.57	5.3	1.7	-0.14	19	16	9.6
	M4	11	13	7	12	12	11	20	9.9	11	9.1	5	6.8	5.1	12	6.9	4.1	9.9	5.3	13
	M5	11	12	9.6	13	10	11	18	11	13	13	11	5.2	3.1	14	14	10	13	4.4	11
	M6	15	13	17	17	15	16	13	16	13	10	6	4.4	3.5	12	11	7.4	3.7	12	11
	M7	8.7	13	6.6	9.1	12	8.4	15	15	16	11	2.3	0.59	0.33	2.8	4.3	1.4	10	8.7	2.4
	M8	11	8.3	9.9	12	14	15	16	8	11	8.2	0.91	0.03	1	2.4	3.9	1.3	8	7.7	0.58
	M9	3.7	3.4	4.5	5.7	3.9	3.5	4	4.6	4.4	3.3	2.5	2.2	1	3.4	5.3	3.3	3.2	2.3	2.8
	M10	9.1	9.4	10	15	8.9	9.1	16	12	12	9.8	9.4	6.4	3	15	13	7.9	15	5.6	11
	M11	1.2	1.7	2.8	2.9	1.3	1.4	-0.84	1.1	2.8	0.94	1.4	0.3	0.93	0.6	0.93	0.12	0.7	-0.87	2.6
	M12	22	30	19	18	23	25	35	35	30	21	6.5	1.2	0.91	20	24	17	28	28	4.4
	M13	1.8	3.2	3	2.2	1.1	1.5	0.47	1.1	5	2.1	0.84	0.46	0.22	1.9	0.64	-0.1	1.1	0.29	3.3
	M14	19	15	6.5	5.1	19	18	19	20	16	14	1.7	4.4	2.3	6.8	6	3.8	6.9	11	6.4
	M15	5.7	11	8.4	7.9	11	9.9	10	7.4	9.5	8.9	9.3	5.7	5.7	17	19	4.9	4.4	5.3	7.4
	M16	5.7	2.7	5.7	2.5	4.9	4.7	6	8.2	3.5	3.4	3.1	6.9	4.2	8	7.8	5.2	5.2	11	11
	M17	-0.27	-0.34	-1.2	-0.91	-0.73	-0.34	0.5	-0.06	0.48	-0.79	-0.17	-0.24	0.65	-0.31	-0.8	-1.2	2.8	-0.92	-0.23
	M18	8	9.4	3.1	4.7	9.5	7.7	7.7	8.6	12	10	0.78	1.1	0.13	1.7	1.7	0.1	11	6.2	3.1
	M19	3.3	2.5	6.8	8.3	4.5	5	6.2	6.7	6.4	1.4	5.6	7.4	2.6	12	9.3	6.7	7	3.2	9.2
	M20	0.78	1.5	4.7	4.7	4.6	3.1	4.9	2.1	2.2	2.8	1.6	3.5	4.9	5.5	5.2	5.1	2.7	1.5	7.1
	M21	7.2	5.1	6.4	3.6	6.3	6.5	6.4	9.2	5.6	4.2	1.7	6	7.9	5	6.5	5.5	3.7	7.8	7.9
	M22	-0.03	-0.66	2.1	2.1	-1.7	-0.84	1.8	0.34	0.8	-0.39	0.9	-1.2	-0.22	-0.17	-1.2	-1.3	3.8	0.81	0.55
	M23	20	18	16	18	21	23	16	23	21	17	6.8	5.1	2.6	9.1	10	5.5	4.5	12	11
	M24	21	31	15	20	40	26	58	59	39	29	1	1.1	0.96	3.1	8.4	1	56	36	3.4
	M25	2.5	9.2	6.9	8.2	4.7	7	6.3	8.3	5	3.4	5.7	7.6	6	11	9	7.3	6	3.7	8.3
	M26	13	9.3	2.8	2.8	16	15	12	6.2	7.8	8.9	0.87	-0.71	0.17	2.8	1.8	1.1	5.7	8.5	6.3
	M27	0.69	2.7	3.8	3.6	0.29	1.2	0.22	1.5	3.7	1.9	4.7	6.7	6.9	5.9	6.4	5.9	5	0.78	6.4
	M28	11	15	2.5	3.8	10	11	15	17	16	16	1.9	0.33	2.1	3.3	3.8	0.53	9.5	9.6	3.6
	M29	15	16	7.7	9.8	16	15	8.7	8.9	18	14	2.5	1.9	0.25	5	5.5	2.7	2.9	6.4	14
	M30	44	48	15	15	50	47	45	42	38	41	0.36	1.1	1.5	2.1	3.2	1.6	13	24	12
	M31	11	12	5.9	4.9	13	8.1	17	7.5	11	8.1	4.1	5.2	6	8	7	4.9	5.3	9.9	5.5
	M32	2.5	2.1	3.6	4.6	4.9	2.1	3.7	2.7	2.5	2.9	3.4	1.6	1.9	3.4	4.9	1.9	2.1	0.56	1.6
	M33	7.5	7.5	1.7	1.5	6.9	7.3	8.5	5.2	7.4	6.3	1.6	1.1	3.5	1.9	2.4	2.8	2	6.7	3.8
	M34	0.8	1	3.5	4.3	-0.53	-0.6	2.2	0.11	1.2	0.23	0.12	-0.14	1.1	0.26	-0.72	-0.52	5.2	1.2	0.47
DiffCoEx	M0	1.7	-0.77	4.8	8.1	4.5	1.8	8.8	0.87	1.5	1.3	2.6	5.1	5.8	3.9	3.1	4.4	5.9	4.4	5.4
	M1	14	15	7	10	14	17	18	13	14	14	2.8	4.1	3.2	11	4.7	3.8	11	6.9	13
	M2	48	38	36	43	52	53	38	29	42	41	1.1	-0.13	0.2	12	8.1	6.1	40	44	4.6
	M3	5.3	2.7	0.59	-0.12	5.6	5.9	4.4	3.3	2.1	2.6	-0.81	-0.82	-0.06	1.6	1.7	1.6	9.8	15	1.4
	M4	3.9	4	4.5	3.5	4.6	4.2	6.5	4.3	5.8	3.9	1.5	2.8	1.7	5.1	1.6	3.1	4.1	3.8	3.6
	M5	9	6.6	2	1	8.6	8.3	4	8.7	3.8	5.1	1.5	0.28	-0.81	3	0.91	1.3	3.9	2.8	1.4
	M6	18	30	5.6	10	37	29	52	47	36	29	0.56	0.77	0.26	6.9	9.8	2.6	51	37	3.5
	M7	-0.15	26	-0.77	0.68	1.2	0.67	20	27	7.1	2.2	-0.28	1.4	-0.09	1.3	0.29	0.75	11	12	11
	M8	-0.35	3.7	4.6	7.7	3.1	1.6	2.1	0.7	5.1	5	-1.2	-0.22	-0.97	2.4	0.98	1.5	2.2	0.58	0.05
	M9	24	19	17	16	22	24	21	25	19	18	2	4.1	6.7	6.7	11	6	9	16	7.2
	M10	4.7	6.9	7.3	7.2	5	5.5	5.1	5.3	7.1	3.7	6.1	7	5.6	11	8.2	6.7	6.2	0.86	9.9
	M11	12	9.1	15	18	12	13	8.4	13	13	7.6	6.3	3.8	2.1	7.7	11	6.7	3.1	7.3	9.7
	M12	43	45	15	15	43	39	40	35	36	37	1.5	0.71	3.5	1.2	3.4	1.5	9.5	37	10
	M13	24	33	25	20	24	29	42	44	34	22	8	0.68	-0.14	26	27	16	48	29	5.5

Supplementary Figure 3: Gene Set Enrichment Analysis (GSEA) plot of non synonymous *de novo* mutations (nsDNM) in patients ascertained for epileptic encephalopathy (EE) for genes coding for proteins with higher degree in the M30 protein-protein interaction (PPI) network.

The GSEA was performed ranking genes according to the number of intra-modular PPI and showed significant enrichment (Normalized Enrichment Score NES: 2.4, FDR q-value: 0.0013).

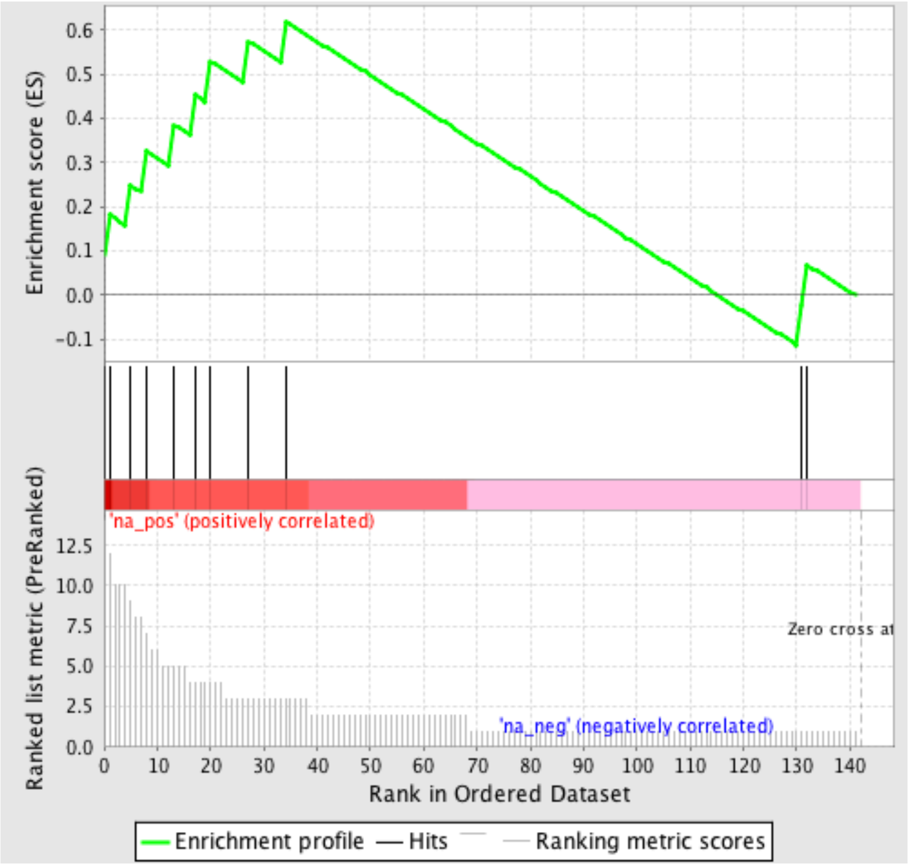

Supplementary Figure 4: Differential expression analysis of M30 genes in epileptic hippocampus in three epilepsies.

a. "Volcano plot' of statistical significance against fold change between cases and controls. Each dot represents one gene of M30. Red dots are genes with non synonymous *de novo* mutation (DNM) in patients ascertained for EE. b. GSEA plots showing significant enrichment of M30 in down-regulated genes in epileptic hippocampus in all three epilepsies (human TLE: NES=-4.51, $P<10^{-5}$; pilocarpine TLE mouse model: NES=-5.83, $P<10^{-5}$; Dravet model: NES=-3.69, $P<10^{-5}$).

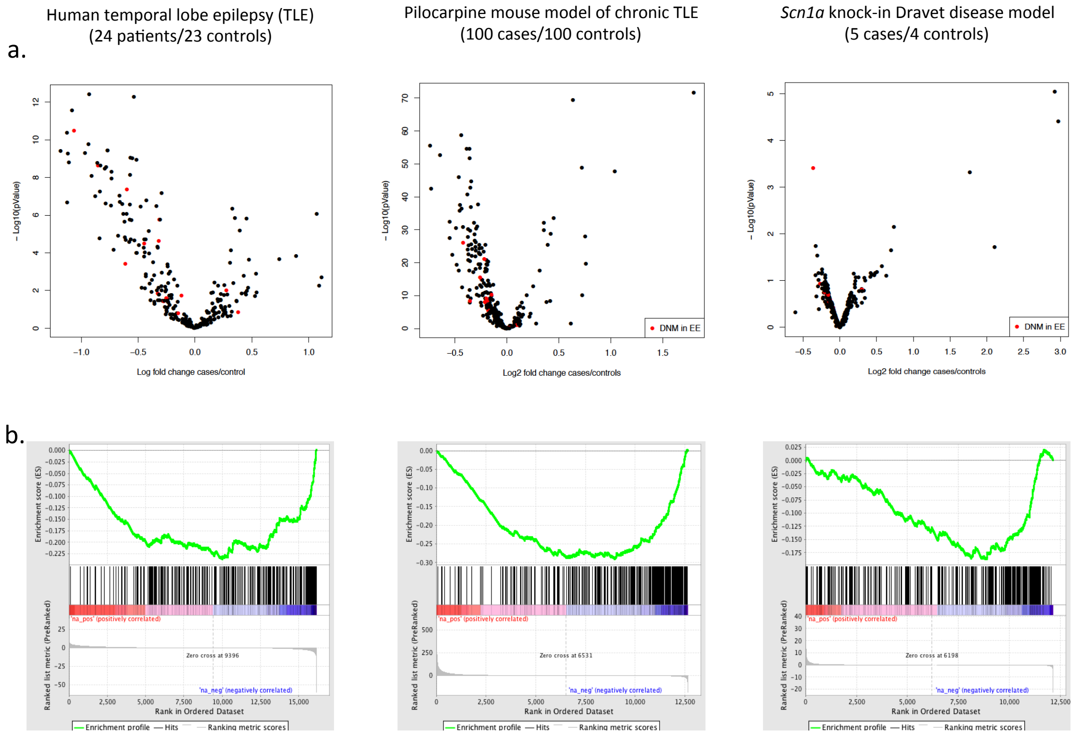

Supplementary Figure 5: GSEA plot of M30 for genes anti-correlated to seizure frequency in the pilocarpine mouse model of chronic TLE.

The GSEA was performed ranking genes according to their Spearman correlation coefficient with frequency of seizures weighted by significance and showed enrichment of M30 in genes anti-correlated to seizure frequency (NES=-6.68, $P < 10^{-4}$).

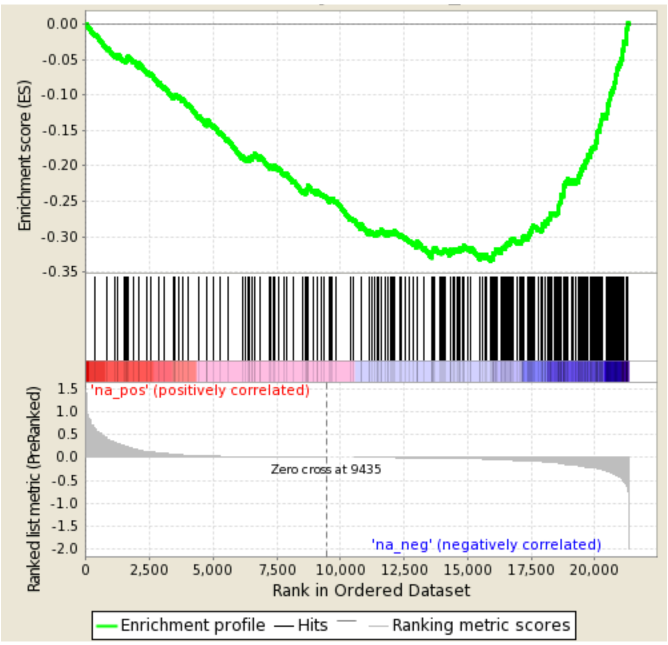

Supplementary Figure 6: Dose effect on significance of enrichment of M30 for genes significantly upregulated by valproic acid.

FET= Fisher Exact Test

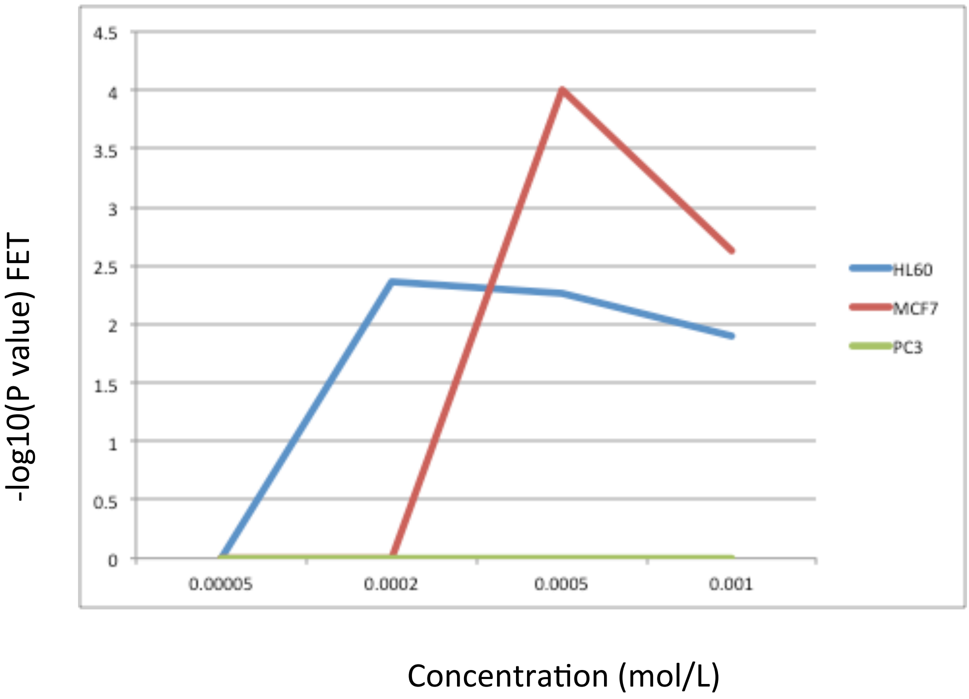

Supplementary Figure 7: Mapped M30 genes which are up or down regulated by valproic acid.

Log2 fold change in a gene's expression on the x-axis plotted against – log10 pvalue of differential expression on the Y. Genes to the left of zero are down-regulated by the drug, and genes to the right are upregulated. Genes significantly differentially expressed (DE) at FDR <10% are highlighted in red.

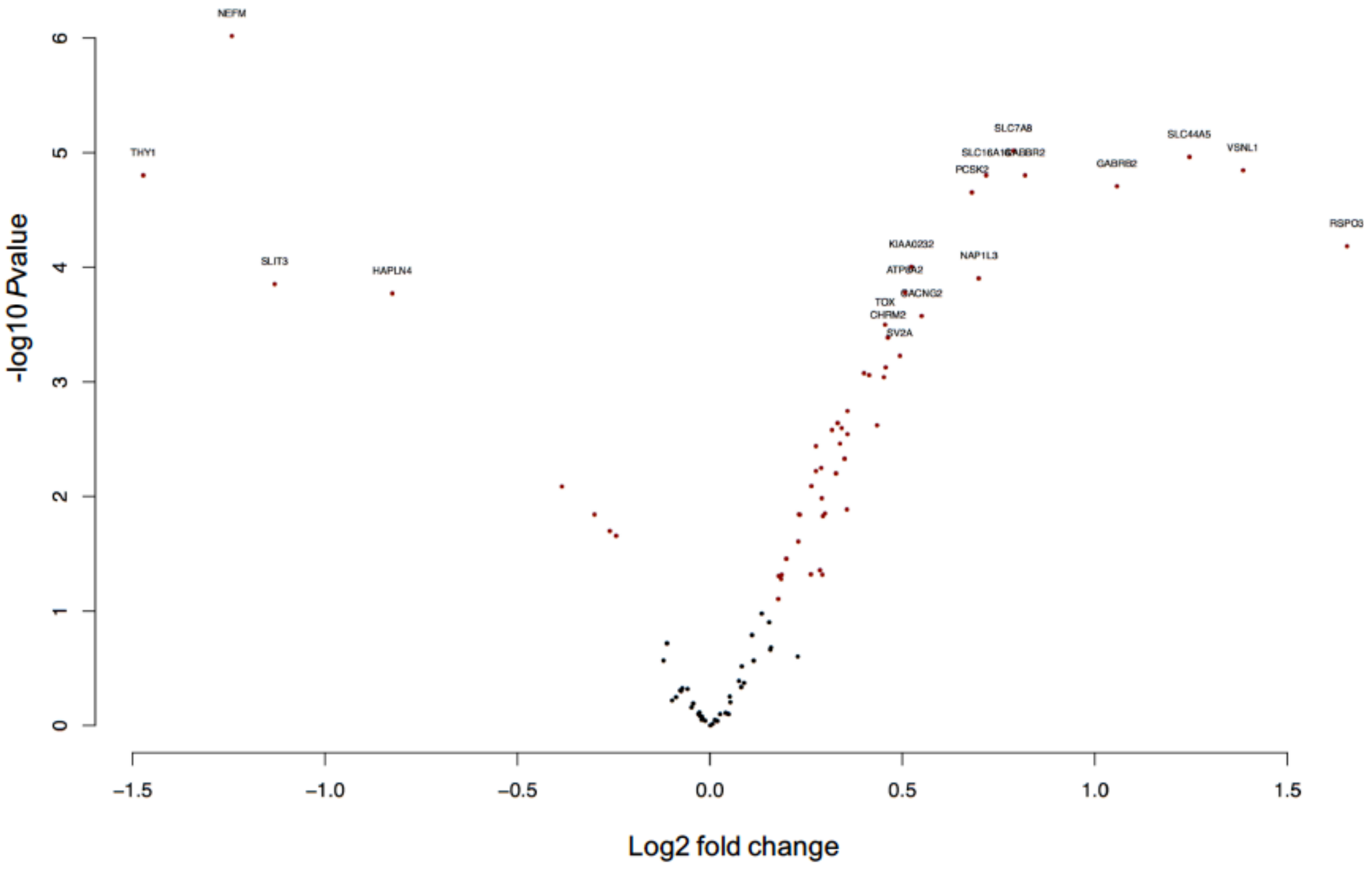

Supplement: Additional file 3: — All additional figures. (PDF 661 kb) [file 13059_2016_1097_MOESM3_ESM.pdf]
